# Whole-Body [^18^F]-Fluoride PET SUV Imaging to Monitor Response to Dasatinib Therapy in Castration-Resistant Prostate Cancer Bone Metastases: Secondary Results from ACRIN 6687

**DOI:** 10.3390/tomography7020013

**Published:** 2021-04-25

**Authors:** Mark Muzi, Finbarr O’Sullivan, Timothy G. Perk, John P. Muzi, David A. Mankoff, Robert Jeraj, Fenghai Duan, Evan Y. Yu

**Affiliations:** 1 School of Medicine, University of Washington, Seattle, WA 98195, USA; jpmuzi@uw.edu (J.P.M.); evanyu@uw.edu (E.Y.Y.); 2 School of Mathematical Sciences, University College, T12 XF62 Cork, Ireland; f.osullivan@ucc.ie; 3 AIQ Solutions, Madison, WI 53717, USA; perk@wisc.edu; 4 Department of Medical Physics, University of Wisconsin, Madison, WI 53705, USA; rjeraj@wisc.edu; 5 Perelman School of Medicine, University of Pennsylvania, Philadelphia, PA 19104, USA; David.Mankoff@pennmedicine.upenn.edu; 6 Department of Biostatistics, Brown University, Providence, RI 02912, USA; fduan@stat.brown.edu

**Keywords:** ACRIN 6687, metastatic castration-resistant prostate cancer (mCRPC), bone metastases, [^18^F]-Fluoride, PET, dasatinib, progression-free survival (PFS), Quantitative Total Bone Imaging (QTBI)

## Abstract

ACRIN 6687, a multi-center clinical trial evaluating differential response of bone metastases to dasatinib in men with metastatic castration-resistant prostate cancer (mCRPC), used [^18^F]-fluoride (NaF) PET imaging. We extend previous ACRIN 6687 dynamic imaging results by examining NaF whole-body (WB) static SUV PET scans acquired after dynamic scanning. Eighteen patients underwent WB NaF imaging prior to and 12 weeks into dasatinib treatment. Regional VOI analysis of the most NaF avid bone metastases and an automated whole-body method using Quantitative Total Bone Imaging software (QTBI; AIQ Solutions, Inc., Madison, WI, USA) were used. We assessed differences in tumor and normal bone, between pre- and on-treatment dasatinib, and evaluated parameters in association with PFS and OS. Significant decrease in average SUV_max_ and average SUV_peak_ occurred in response to dasatinib. Univariate and multivariate analysis showed NaF uptake had significant association with PFS. Pharmacodynamic changes with dasatinib in tumor bone can be identified by WB NaF PET in men with mCRPC. WB PET has the benefit of examining the entire body and is less complicated than single FOV dynamic imaging.

## 1. Introduction

Approximately 70% of men with advanced prostate cancer harbor osteoblastic bone metastases [1]. Imaging of bone metastases typically relies on bone scintigraphy and anatomic modalities such as CT and MRI. However, these methods measure qualitative changes in bone turnover (bone scan) or bone structure (MRI, CT) but not direct metastatic tumor cell activity. Clinically meaningful prostate cancer treatment response has been difficult to define quantitatively, as there is no uniformly accepted surrogate marker that correlates with long-term outcomes to optimally guide patient management and new drug development.

The use of positron emission tomography (PET) to monitor response to therapy in prostate cancer is inherently quantitative. PET can measure in vivo tumor and normal tissue biology using tracers to map many metabolic pathways, including bone osteoblastic metabolism using [^18^F]-fluoride (NaF) PET [2,3]. NaF PET offers a quantitative measure of osteoblastic bone formation and remodeling, and is appropriate for imaging the blastic lesions observed in prostate cancer [4]. Additionally, when compared to standard ^99M^Tc-based bone scintigraphy, NaF PET offers improved sensitivity of detection and when combined with CT, specificity is also improved [5,6,7].

ACRIN 6687 was a prospective, multi-center phase 2 trial that used NaF PET to probe the response of dasatinib (SPRYCEL^®^; Bristol-Myers Squibb) treatment, a SRC kinase inhibitor that decreases bone turnover, in men with metastatic castration-resistant prostate cancer (mCRPC) [8]. The trial was designed to evaluate differential response of normal and tumor bone to dasatinib treatment using NaF PET using a protocol that began with dynamic single field-of-view (FOV) imaging and then was followed by static whole-body (WB) scans with multiple FOVs. Previous kinetic modelling results from single FOV dynamic imaging found significant differences in changes of the PET kinetic parameters from tumor bone compared to normal bone in response to dasatinib treatment. Changes in the 30–60 min summed SUV metrics from the dynamic acquisition had a modest association (*p* = 0.056, *n* = 12 patients) with progression-free survival (PFS), where progression was determined by the Prostate Cancer Working Group 2 (PCWG2) [9] criteria.

Although the initial results for the ACRIN 6687 trial were intriguing, we recognize the potential limitations of dynamic single FOV analyses for general use and widespread adoption. Specifically, although dynamic studies may offer breadth of analysis, the level of complexity and lack of standardization are not practical for broad utilization. In the initial set of analyses using the 30–60 min SUV images, changes in the average SUV_max_ for up to 5 tumors (SUV_maxavg_) in a patient not only had significant differential changes to dasatinib therapy in tumor vs. normal bone, but those changes had marginal association with progression free survival (PFS); these were features not displayed by dynamic Ki (metabolic flux) or K_1_ (tracer transport) kinetic parameters. This lends further credence to the concept of simplifying the NaF PET image analysis with SUV only. Additionally, the previously reported limited FOV may have omitted important information from metastatic lesions outside of the single FOV. As part of a post-hoc analysis not proposed in the original ACRIN 6687 clinical trial, we sought to determine if important information obtained from outside of the dynamic FOV could offer additional clinical and prognostic information, comparable and/or incremental to earlier published dynamic data. Previous reports using WB fluoride analysis also showed a relationship of SUV measures to PFS for patients that received either a docetaxel-based chemotherapy regimen or an androgen receptor pathway inhibitor [10]. Here we examine SUV analysis results from multi-FOV WB static NaF PET imaging scans, acquired after a one-hour dynamic scan, in mCRPC patients recruited to ACRIN 6687 at baseline and after receiving 12 weeks of dasatinib treatment. Statistical analysis of the clinical and PET imaging data was undertaken in order to identify potentially interesting associations between various biomarkers (PET and blood borne) and patient outcomes. As is the nature of secondary investigations, the reported data analysis and relationships cannot be interpreted in the same way that the analysis for the primary hypothesis of the underlying clinical trial that has been reported [8].

## 2. Materials and Methods

### 2.1. Study Design

Study design and treatments (Supplementary Materials, Figure S1), patient eligibility, imaging protocol, regulatory approval, radiochemistry and study endpoints have been previously described [8]. Briefly, American College of Radiology Imaging Network (ACRIN) 6687 was a phase 2 trial conducted by ACRIN at 4 Prostate Cancer Clinical Trials Consortium (PCCTC) centers: University of Washington, Duke University, Oregon Health Sciences University and the Dana-Farber Cancer Institute (NCT00936975). ACRIN 6687 protocol was approved at each site’s institutional review board and other local regulatory agencies. Informed consent was obtained from all individual participants included in the study prior to trial enrollment. Patients enrolled on the study had to have metastatic castration-resistant prostate cancer with at least one convincing bone metastasis defined by bone scintigraphy, CT scan or plain X-ray. All patients eligible for ACRIN 6687 were first enrolled in a clinical trial (NCT00918385) where patients were selected either for nilutamide or dasatinib based on a 300-gene signature found on a metastatic biopsy. Only patients receiving dasatinib were imaged on ACRIN 6687. The PET imaging protocol included a single field-of-view (FOV) low-dose CT scan for attenuation correction, a one-hour dynamic PET emission scan consisting of 45 time frames over 60 min immediately following the NaF injection, a multiple FOV (range 5–7 FOV) WB PET emission scan from base of skull to mid-thigh and a multiple FOV WB low-dose CT scan. Eligible patients with bone mCRPC underwent the WB NaF PET scan, that occurred starting at approximately 75 min (range 53 to 95 min) after NaF injection, with an on-average mid scan time of 90 min (range 64–110 min) prior to and 12 weeks after the onset of treatment with dasatinib therapy to determine if the nature of the drug effect could be ascertained through PET/CT imaging. The WB scans were approximately 25–40 min in duration, making the uptake time range from the start to the end of the scan 53–130 min after injection with a mean mid scan time of 90 min. Individual patient WB PET scan acquisition parameters and reconstructed image resolution values appear in Supplementary Materials Table S1 and the WB time profile in Figure S2. Additional scanning before and while on dasatinib treatment included a clinical CT scan and a ^99m^Tc-methylene diphosphonate (^99m^Tc-MDP) bone scan.

### 2.2. Clinical Assessments

Patients were clinically followed after initiation of dasatinib with clinical visits every 4 weeks and repeated bone and CT scan imaging every 12 weeks until radiographic or clinical progression, significant toxicity necessitating cessation of dasatinib or until patient withdrawal from the trial. Baseline pathologic Gleason grade scores from archival prostate tissue was obtained from local participating sites. Biomarkers from blood and urine samples obtained at baseline and throughout therapy included bone alkaline phosphatase (BAP), an indicator of bone metabolism, urine N-telopeptide (uNTX), an indicator of bone resorption and prostate specific antigen (PSA), an indicator of progressive disease.

### 2.3. PET Image Analysis

Unlike the previous single dynamic FOV investigation surrounding the pelvis and lower spinal column, WB image analysis used both a traditional lesion-level volume-of-interest (VOI) method and a whole-body patient-level assessment. The lesion-level VOI method collected up to 5 of the most NaF avid bone metastases using the highest NaF SUV_max_ uptake value, the maximal voxel within a 1cc VOI, on the baseline scan. The average activity from a 1cc spherical VOI placed over the hottest region of the tumor as described in the PERCIST protocol [11] was the SUV_peak_. These VOIs were placed in the same anatomical location on the mid-therapy scan. Based on previous reports, only tumors with a SUV_peak_ threshold of 15 g/mL were included in the analysis [10,12]. Although Kurdziel et al. used a segmentation SUV threshold of 10 g/mL [13], a later study by Rohren et al. showed that lesion ROIs identified using this threshold still included normal bone activity [14]. Lesions smaller than 1.5 cc as measured by PET volume were also excluded.

Each selected tumor region from the NaF PET and corresponding CT images was reviewed by an experienced PET image nuclear medicine radiologist and a prostate cancer oncologist and confirmed as malignant. Bone lesion VOIs, along with matched areas of normal bone, were used for intensity analysis by SUV_max_ and SUV_peak_. Tumor assessments were performed using the average of up to 5 tumors from each patient, using the notation from the original ACRIN 6687 report of SUV_maxavg_ and SUV_peakavg_ and the index lesion SUV_max_ for each patient. The index lesion was the single lesion with the most NaF avidity. These SUV metrics have been shown to be useful in prior studies of NaF PET imaging of mCRPC patients for evaluation of treatment response [8,10,15,16]. Tumor-matched normal bone regions identified by both CT and NaF PET of identical volume to tumor regions were also constructed.

### 2.4. Quantitative Total Bone Imaging (QTBI) Analysis

Whole-body patient-level image analysis utilized the bone metastases software application from the University of Wisconsin and AIQ Solutions (Madison, WI, USA) [12,17]. Briefly, CT images were segmented into skeletal regions using an atlas-based segmentation approach [18], then region-specific optimized thresholds were used to detect lesions [19] on the PET image. Following lesion detection, a random forest model and manual review were applied to exclude lesions that were likely to be benign [20]. Patient-level PET parameters used for tumor assessment included qSUV_max_, qSUV_peak_, qSUV_total_ (total tumor burden) and qVF (volume fraction), where the q indicates that the parameters are derived from QTBI analysis. The peak SUV was determined by placing 1cc spheres on each of the 5 lesions with the highest SUV_max_ and averaging the extracted values.

### 2.5. Statistics

Pre- and on-treatment differences of lesion-level and patient-level PET parameters were assessed using standard paired t-tests. Repeatability studies showed that SUV_max_ from lesion level analysis coefficient of variation was 14.1%, while at the patient level, it was slightly smaller: 12.0% [12]. Variation of other PET SUV metrics from repeatability analysis were similar in magnitude. While the data have limited power to properly verify normality of the underlying data, the Shapiro-test of normality showed little evidence of departure from such an assumption. *p*-values obtained by the t-test were found to be in close agreement with those obtained using a non-parametric Wilcox rank test approach. Pairwise comparisons were summarized in terms of rank correlations. Rank correlation was used because it has the ability to evaluate monotone relations, not just linear ones. Note that overall, 96 separate *p*-values were generated in this analysis. It is important to appreciate that the *p*-values reported are without adjustment for multiple comparisons. Our justification for this is that results presented are not offered as definitive resolutions to the 96 hypotheses being considered, instead they are presented as a way to guide the selection of a much more limited set of hypotheses that might merit further investigation in a prospective clinical trial. See the discussion for further comment.

Pre-treatment PET values and the change from pre- to on-treatment PET values were evaluated in association with PFS and overall survival (OS), both of which are continuous variables measured in days from the onset of dasatinib treatment. PFS is determined as the number of days from dasatinib treatment to the first progression event as evaluated by PCWG2 criteria. The relationship between PET parameters and outcome measures of PFS was evaluated by univariate and multivariate regression analysis. The overall survival (OS) data are incomplete (censored) and so a Cox proportional hazards model was used for both univariate and multivariate analysis.

For multivariate regressions, we report *p*-values associated with the PET parameter combined with the base model. This approach assesses the additional prognostic contribution of the PET parameter, after adjustment for a base model of established clinical variables; age and baseline ln(BAP). The *p*-values reported for the SUV variables in multivariate analysis assess the added impact of the SUV variables in a context where there is adjustment (by the multivariate method) for the ln(BAP) and age covariates. These are not *p*-values for the overall model. The multivariate analysis gives a more precise appreciation of the ‘added-benefit’ of the PET information. In the case of OS, the effects of the PET variables are reported in terms of the excess risk, or hazard ratio (HR), associated with a 1-SD change in the PET variable.

As a result that the PFS data were complete (no censoring), the relation between prognostic factors, such as age, BAP or PET variables, and PFS was analyzed by multiple linear regression. Cox modeling analysis was also considered, but given the more precise nature of regression analysis, the multiple regression analysis was used in this report. Given the limited sample size and the consequent concerns regarding the adequacy of standard asymptotic Gaussian approximations for inferences, Efron’s Bootstrap [21] with 500 replicates was used in multivariate outcome analysis.

Effects were assessed using a two-sided z-test based on the bootstrap estimated mean and standard error (SE) values. Additionally, changes in PET uptake parameters in response to dasatinib treatment, were compared with changes in markers of bone turnover, urinary N-telopeptide (uNTX), bone alkaline phosphatase (BAP) and PSA using Kendall’s tau-b correlation. All statistical tests were performed in R, and acronyms are defined in Table 1.

## 3. Results

### 3.1. Patients

Of the 18 patients enrolled in the trial (median age 69 years range 48–86), one withdrew from the study with no follow-up on PFS or OS after the first PET scan, and was excluded from this analysis leaving 17 evaluable patients for WB PET baseline imaging. Three patients, with worse baseline prognostic features, did not undergo an on-treatment PET imaging study due to clinical progression while on dasatinib; this resulted in early discontinuation from the trial prior to the second imaging time point. In our initial ACRIN 6687 publication on dynamic imaging results, two studies were omitted due to technical issues with the dynamic scan, but their WB scan was useable for this analysis and therefore were included. Seventeen patients had either met progression criteria or death by the time of this investigation. Thus, 14 patients had evaluable pre- and on-treatment dasatinib WB NaF PET imaging. The baseline patient and PET imaging characteristics appear in Supplemental Materials Table S1.

### 3.2. PET Findings

**Figure 1:**
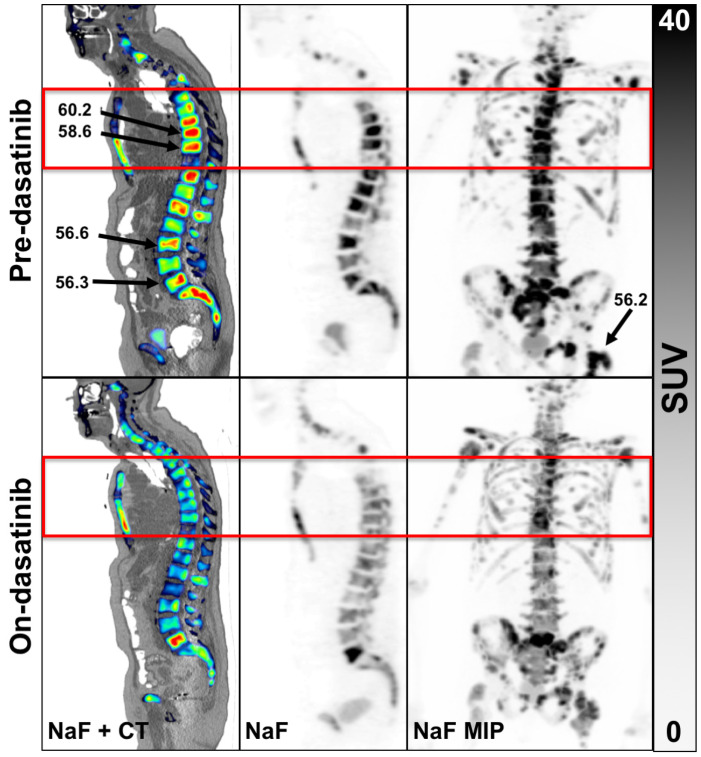
Example of an 82-year-old patient scan 1 (**top** row) and scan 2 (**bottom** row). Panels left to right, NaF overlaid on CT, NaF alone and with NaF PET maximal image projection (MIP) of the entire WB volume. The red box is the single FOV for the dynamic scan. Three of the 5 hottest tumors were not located in the single dynamic FOV, the results of which were reported previously [8]. An example WB patient with none of the hottest tumors in the dynamic FOV appears in Supplementary Materials Figure S3.

**Figure 2:**
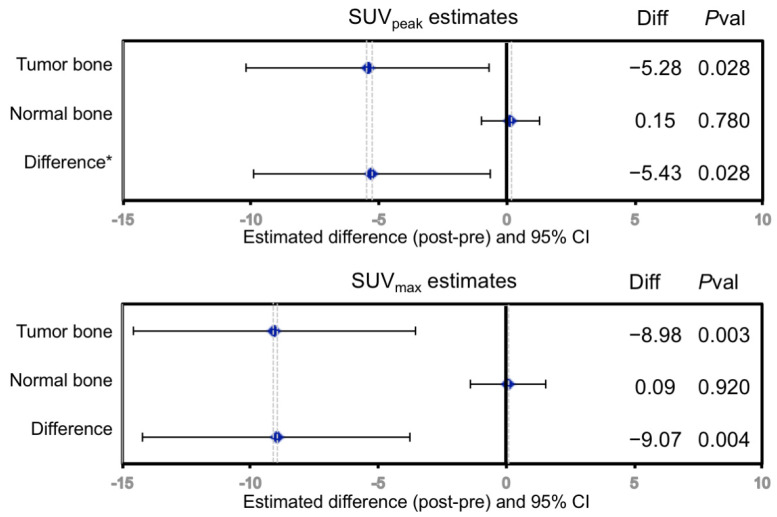
Change in regional ^18^F uptake in response to dasatinib treatment in mCRPC bone metastases measured by SUVpeak, SUVmax. No significant changes were seen in normal bone. Diff = Scan2—Scan1; *P*val = *p*-value. * Difference = Δ in tumor bone—Δ in normal bone.

In the original single FOV report for ACRIN 6687 [8], up to 5 bone lesions were selected by the local site physician, which occurred in the pelvis or along the lower spinal column. The WB tumor selection criteria at the lesion level presented in this report was determined by averaging up to 5 of the highest NaF SUV_max_ uptake bone lesions using a lower threshold of 15 g/mL. However, only 19 WB regions of the 70 dynamic regions (27%) overlapped between the dynamic and static PET series (Figure 1). Thus, many of the hottest lesions from the WB SUV images acquired at an average mid-acquisition time of 90 min after injection were not present in the single FOV SUV image acquired precisely at 45 min (30–60 min summed SUV) from the dynamic series. A summary of the lesion-level PET parameter values before and while on-dasatinib treatment appear in Table 2. Individual patient NaF PET SUV uptake values appear in Supplementary Materials Table S2. The average uptake values for all tumors from a patient study were represented as SUV_peakavg_ and SUV_maxavg_, while the values for the hottest single index lesion from each patient were represented as index SUV_max_ and index SUV_peak_. Fifteen of the 17 evaluable patients had 5 tumor sites above the threshold, while 1 patient had 2 tumor sites and 1 patient had 1 tumor site above the SUV_peak_ threshold of 15 g/mL. Significant average decreases were observed in SUV_maxavg_ (−20% ± 12% 95%CI, *p* = 0.001), SUV_peakavg_ (−17% ± 14% 95%CI, *p* = 0.013), index SUV_max_ (−16% ± 14% 95%CI, *p* = 0.025) and index SUV_peak_ (−16% ± 15% 95%CI, *p* = 0.049) in bone metastases in response to dasatinib, while no significant change was observed in normal bone (Figure 2). Significance was based on repeatability results of NaF in mCRPC patients [12]. Significant differences in changes from tumor bone compared to normal bone in response to dasatinib were noted for SUV_maxavg_ (*p* = 0.004) and SUV_peakavg_ (*p* = 0.028).

Results of patient-level QTBI analysis used only 15 patient scans at baseline and 12 s time point on-treatment scans due to technical issues related to image scaling and image quality for 2 patients between the dual time point scans. No significant change between pre-dasatinib and on-treatment NaF uptake for qSUV_max_, qSUV_peak_, qSUV_total_ and VF was observed for the 12 patients (Table 1). Individual patient-level QTBI uptake values appear in Supplementary Materials Table S3 with a patient example analysis in Supplementary Materials Figure S4.

### 3.3. Statistical Analyses

In the case of progression, the data are complete (no censoring) so standard multiple linear regression analysis was used. However, for OS, 3 patients were censored of the 17 evaluable patients and a Cox proportional hazard model was applied to account for censoring.

In univariate analysis of PET variables as predictors of PFS (Table 3), only elevated baseline qSUV_total_ and baseline qVF were significantly associated with PFS (*p* = 0.023 and *p* = 0.011, respectively), where higher values lead to earlier progression. There was no clear association of the change in any other lesion-level or patient-level PET parameter with PFS or OS for univariate analysis unlike the univariate analysis results of the original ACRIN 6687 report that showed a borderline correlation of change in SUV_maxavg_ to PFS (*p* = 0.056). Bootstrap results are not reported for univariate analyses, but they are provided in Supplementary Materials Table S4.

In multivariate analyses (Table 4), the regression model included age, the logarithm of baseline bone alkaline phosphatase (ln(BAP)) and the PET parameter as covariates. Age and ln(BAP) were found to be strong predictors of disease progression in univariate analysis [8]. Baseline lesion-level SUV_maxavg_ and SUV_peakavg_ values from the 17 patients showed an association with PCWG2 PFS (*p* = 0.043 and *p* = 0.018, respectively) using multivariate analysis. The multivariate analysis used for QTBI parameters had the same base model of age and ln(BAP) described above, and showed that baseline qSUV_peak_ also had a significant relationship with PFS (*p* = 0.025). The multivariate analysis showed no relationships to OS for any PET parameter at baseline or change in the parameter while on-dasatinib. The original report for ACRIN 6687 [8] did not perform multivariate analysis.

The actual PFS versus the predicted progression based on multivariate regression analysis is shown in Supplementary Materials Figure S5. The predicted progression relies on the multivariate base model that includes the covariates of age and baseline ln(BAP) with the addition of a PET parameter and shows a high correlation (r = 0.83) between the actual and predicted progression (*p* = 0.001).

Changes in patient and lesion-level NaF PET uptake parameters in response to dasatinib in bone metastases to the change in PSA and bone biomarkers appear in Table 5. Specifically, change in BAP had a significant negative correlation with baseline NaF PET assessed by lesion-level SUV_peakavg_ and SUV_maxavg_. Universally, PET uptake parameters decreased from before to while on-dasatinib treatment, while BAP levels increased or stayed the same. Change in uNTX was correlated to the SUV_max_ of the index lesion, but no other PET variables. PSA had no correlation with changes in any NaF PET uptake values.

## 4. Discussion

Similar to the results in our previous report of ACRIN 6687 evaluating a limited dynamic FOV, NaF PET WB uptake also reveals the distinct patterns of pharmacodynamic changes in bone mCRPC from normal bone in response to therapy with dasatinib, as displayed in Figure 2. There appears to be a differential effect of dasatinib on normal compared to tumor bone in men with mCRPC, as measured by fluoride uptake and fluoride bone incorporation.

The previous ACRIN 6687 report [8] showed that SUV_maxavg_ from a single FOV NaF image summed exactly from 30–60 min had a large decrease in bone mCRPC uptake in response to treatment with dasatinib, and that a decrease in SUV_maxavg_ marginally correlated with shorter PFS (*p* = 0.056), indicating that patients with a lower decline in SUV_maxavg_ had longer PFS. In the current WB lesion-level SUV analysis, baseline or changes in uptake measures collected later, on average WB imaging starting approximately 75 min after injection (range 53 to 95 min), failed to find significance with PFS or OS in univariate analysis. The later WB scan acquired with a mid-scan average of 90 min after injection (range 65 to 110 min) might be different from the single FOV dynamic scan collected precisely at mid-scan 45 min (30 to 60 min SUV image) due to tracer clearance that is independent of the disease, fewer counts with increased noise and the large variability of uptake time between patient WB scans, that all have the effect of increasing variability. The wide range in the time of WB image acquisition from the injection time in this multi-center trial can increase variability in SUV measurements by as much as 25% for 15 min deviations [22,23] and may significantly affect the correlation of WB NaF measures to PFS where uptake times differ by more than 40 min.

The assessment of up to the 5 hottest tumors with a threshold SUV, is similar to prior methods, but may not be as useful as the selection of tumors and imaging FOV by local clinicians that utilized information based on their clinical impression of the patients in the ACRIN 6687 primary aim report [8]. Averaging the SUV_peak_ or SUV_max_ over 5 tumors may capture the intensity, but not the spatial distribution of a tumor and [10,15] therefore may be unable to determine total tumor burden, as the QTBI analysis offers. Using QTBI analysis, Harmon et al. [10] have found that total tumor burden determining a SUV_total_ metric via bone segmentation followed by thresholding the NaF SUV at 15g/mL has been valuable in assessing response in mCRPC patients using an effective therapy, such as androgen receptor pathway inhibitors or a docetaxel-based chemotherapy regimen. Patient-level WB assessment using QTBI software for the patients presented here did show that large baseline total tumor burden (qSUV_total_) and tumor volume fraction (qVF) were significantly associated with shorter PFS in univariate analysis, suggesting that a large, intense tumor burden at baseline indicates poor clinical outcome. However, in univariate analysis the change in patient-level PET parameters from QTBI analysis failed to show a relationship to PFS, and no patient-level parameter showed association with OS.

The inability to observe a definitive relationship between changes in NaF PET uptake and PFS or OS may also be because the effect of dasatinib in mCRPC patients is marginal. Dasatinib has not been successful in demonstrating overall survival benefit in phase 3 trials of men with mCRPC [24]. Although the effects of dasatinib on bone have been clearly documented, it does not appear to offer significant anti-tumor efficacy [25,26]. The lack of association of changes in PET parameters to PFS or OS may be that the disease burden was so high in these mCRPC patients, that any response was buried in either PET measurement variability or dasatinib is an ineffective antineoplastic treatment against mCRPC.

However, a multiple variable statistical model that has covariates of age, a clinical biomarker (baseline ln(BAP)) and an NaF PET uptake measure showed that lesion-level baseline SUV_maxavg_, baseline SUV_peakavg_ and patient-level baseline qSUV_peak_ were all significantly associated with longer PFS in this small cohort (Table 4). No PET parameter used in multivariate modeling analysis showed significant association with OS. The major multivariate model driving component is ln(BAP), which along with age and measures of NaF uptake aids in optimizing the estimates of progression. BAP and NaF uptake are expected to be closely related, as bone turnover (BAP) goes hand-in-hand with new bone formation and matrix mineralization (fluoride uptake on NaF PET). High baseline BAP and high NaF uptake might indicate a more favorable blastic phenotype and longer progression, while baseline BAP and lower NaF might indicate a more lytic phenotype and more aggressive clinical behavior.

The statistical results for the multivariate analysis might be affected by the large variation in image acquisition times between patients (see Supplementary Materials Figure S2), which can increase variability by as much as 75% for over 40 min deviation in uptake time between patient scans [22,23]. Outcomes using NaF PET have been different when more efficacious agents, with proven survival benefit, such as androgen axis inhibiting therapeutics or docetaxel chemotherapy have been used. In prior published studies with a larger cohort of patients (*n* = 56), mid-treatment findings with NaF imaging alone have association with PFS [10]. This suggests that NaF PET imaging has potential for assessment of treatment efficacy of some therapies in men with mCRPC.

Interestingly, we observed a negative correlation between a decreasing change in lesion-level SUV parameters (ΔSUV_maxavg_, ΔSUV_peakavg_, ΔIndex SUV_max_) and an increase in bone alkaline phosphatase (ΔBAP). This relationship was noted in the initial report on the ACRIN 6687 trial that patients with the largest decrease in PET uptake parameters had worse outcome than those that stayed the same or increased [8]. An increase in BAP levels may be due to dasatinib treatment, which has been shown previously to promote osteoblast differentiation [27] and mineralization that could lead to a relative activation and a transient increase in BAP levels indicative of a healing or reparative response [28,29,30]. Increased osteoblastic activity would also be expected to lead to a relative increase of NaF uptake. We did not follow these patients after completion of dasatinib treatment with repeat measurements of BAP, thus it is speculative to associate a decrease in BAP levels in this small cohort of patients with better outcome; however, this finding indicates some mechanistic consistency between prior findings based on dynamic imaging and the current WB analysis. Change in uNTX and PSA had no correlation with changes by NaF PET.

Given the very limited capacity of the dataset (*n* = 17 at baseline, *n* = 14 with an additional scan at 12 weeks into therapy) and the many measurements carried out, there is no real scope to carry out any type of internal cross-validation. The bootstrapping approach used in evaluating the relationship between PET variables and outcomes (PFS and OS) provides more defensible estimates of statistical significance of the reported effects and provides some measure of adjustment for the limited sample size. Nevertheless, our exploratory analysis is mainly offered to provide some guidance on what relationships may be worth future investigation via a prospective clinical trial. The most glaring limitation of this study, however, was the small number of evaluable patients recruited and an even smaller subset that completed the second PET scan during dasatinib treatment, limiting statistical power for prediction of PFS and OS.

## 5. Conclusions

The preferential effect of dasatinib in tumor bone over normal bone is well characterized by static WB imaging using NaF PET before and while on dasatinib treatment, and was largely confirmatory of the dynamic results from these same patients [8]. The association of changes in NaF uptake while on dasatinib treatment and PFS or OS were not evident. Dasatinib had some enhanced targeting to involved disease sites but the impact on the disease overall progression was minimal. However, baseline total tumor burden and tumor volume fraction was predictive of a shorter PFS. We had hoped to observe greater effect on tumor bone, disease progression and overall survival but dasatinib showed limited efficacy as a therapeutic for mCRPC patients.

## Figures and Tables

**Table 1:** Definition of acronyms.

Acronym	Definition
Δ	The difference of a parameter between scan 2 and scan 1
^99m^Tc-MDP	^99m^Tc-methylene diphosphonate
ACRIN	American College of Radiology Imaging Network
AR	Androgen receptor
BAP	Bone alkaline phosphatase
CT	Computed tomography
Diff	The difference of a parameter between scan 2 and scan 1
FOV	Field of view, usually axial
HR	Hazard ratio
index SUV_max_	The hottest baseline lesion (index lesion) SUV_max_ value
index SUV_peak_	The hottest baseline lesion (index lesion) SUV_peak_ value
K1	A model parameter estimating transport of the tracer from blood to tissue
Ki	Metabolic flux determined from the model parameters (K_1_ × k_3_)/(k_2_ + k_3_)
mCRPC	Metastatic castration resistant prostate cancer
MIP	Maximal image projection flattening a 3D image series to 2D
MRI	Magnetic resonance imaging
NaF	^18^F-sodium fluoride
OS	Overall survival
PCWG2	Prostate cancer working group 2
PET	Positron emission tomography
PFS	Progression free survival
Pval	The *p*-value of a comparison between two arrays of data
qSUV_max_	QTBI analysis of SUV_max_, the maximum uptake in the tumor volume (g/mL)
qSUV_peak_	QTBI analysis of SUV_peak_ (g/mL)
qSUV_total_	QTBI analysis of total tumor burden, the sum of voxel SUVs in the tumor volume.
QTBI	Quantitative total bone imaging analysis software, AIQ Solutions, Madison, WI
qVF	QTBI analysis of the tumor volume fraction compared to the total bone volume
ROI	Region of interest
SE	Standard Error
SUV	Standard uptake value
SUV_max_	The maximum SUV voxel within a tumor (g/mL)
SUV_maxavg_	The average of up to 5 tumor SUV_max_ values (g/mL)
SUV_peak_	The average activity of a 1cc spherical VOI over maximal tumor activity (g/mL)
SUV_peakavg_	The average of up to 5 tumor SUV_peak_ values (g/mL)
uNTX	Urinary N-telopeptide
VOI	Volume of interest
WB	Whole-body PET scan

**Table 2:** [^18^F]-Fluoride uptake parameters in bone tumors.

Parameters	Baseline	On-Dasatinib	Change	
	NaF PET	NaF PET	On-Dasatinib	% Change
	(SD)	(SD)	(SD)	(*p*-Value)
^†^ Lesion-level				
SUV_maxavg_ (g/mL)	47.1	38.3	−9.5	−20.1%
	(16.7)	(17.0)	(9.6)	**(0.001)**
SUV_peakavg_ (g/mL)	34.5	28.8	−5.8	−16.2%
	(13.3)	(13.0)	(8.3)	**(0.013)**
Index SUV_max_ (g/mL)	60.0	52.8	−9.8	−14.3%
	(27.3)	(24.9)	(14.7)	**(0.025)**
Index SUV_peak_ (g/mL)	45.8	40.3	−7.1	−12.9%
	(21.6)	(18.8)	(11.3)	**(0.049)**
^‡^ Patient-Level				
qSUV_max_ (g/mL)	61.2	64.5	−2.7	0.3%
	(27.4)	(26.1)	(13.9)	(0.569)
qSUV_peak_ (g/mL)	37.6	37.8	−0.6	2.2%
	(15.7)	(12.5)	(7.0)	(0.470)
qSUV_total_ (g/mL × cc)	8234	7307	576	30.0%
	(8914)	(6950)	(1553)	(0.176)
qVF	9.9	8.8	0.3	25.8%
	(10.2)	(8.3)	(0.5)	(0.120)

^†^ Lesion-level average results and average change for 17 patient values at baseline and 14 patients that were scanned while on-dasatinib with standard deviation below in parentheses. Lesion-level Index is the single hottest lesion for the patient. ^‡^ Patent-level parameters from QTBI analysis, indicated by a q preceding the parameter, was performed on 16 patients at baseline and 12 while on-dasatinib. Patient-level qVF is the volume fraction of the tumor from QTBI analysis. Boldface type indicates a significant (*p* ≤ 0.05) decrease in the PET value from baseline.

**Table 3:** Univariate analysis of PET variables to PFS and OS (*p*-values, HR).

PET Parameter	PFS	OS	HR
^†^ Lesion-Level			
SUV_maxavg_1	0.549	0.547	1.199
ΔSUV_maxavg_	0.836	0.253	0.659
SUV_peakavg_1	0.437	0.494	1.229
ΔSUV_peakavg_	0.622	0.443	0.765
Index SUV_max_1	0.631	0.726	1.112
Index ΔSUV_max_	0.760	0.407	0.739
Index SUV_peak_1	0.630	0.678	1.128
Index ΔSUV_peak_	0.884	0.336	0.716
^‡^ Patient-Level			
qSUV_max_1	0.850	0.745	1.101
ΔqSUV_max_	0.780	0.634	0.848
qSUV_peak_1	0.553	0.454	1.285
ΔqSUV_peak_	0.781	0.485	0.787
qSUV_total_1	**0.023**	0.061	1.884
ΔqSUV_total_	0.889	0.260	0.668
qVF1	**0.011**	0.104	1.687
ΔqVF	0.680	0.704	0.869

^†^ The lesion-level analyses were performed on up to 5 tumors per patient selected by uptake intensity for 17 patients at baseline. The change (Δ) while on-dastinib was determined on 14 of the 17 patients. The PFS column has the *p*-value for the PET parameter in analysis of PCWG2 progression free survival. The OS column has the *p*-value for the PET parameter in the analysis of overall survival, and HR has the associated hazard ratio corresponding to a 1-SD increase in the PET parameter. ^‡^ The patient-level whole-body QTBI analyses were performed on 16 patients at baseline, while change was determined on 12 of the 16 patients. Boldface type indicates a significant (*p* ≤ 0.05) association with outcome.

**Table 4:** Multivariate analysis of PET variables to PFS and OS (*p*-values, HR).

^†^ PET Parameter	PFS			OS		
	Days	SE	*p*-Value	HR	SE	*p*-Value
Lesion-Level						
SUV_maxavg_1	26.5	13.1	**0.043**	1.135	0.856	0.875
ΔSUV_maxavg_	−2.1	21.5	0.923	0.800	1.081	0.853
SUV_peakavg_1	32.0	13.5	**0.018**	1.421	2.770	0.879
ΔSUV_peakavg_	−10.4	21.9	0.635	1.142	3.486	0.968
Index SUV_max_1	17.3	14.2	0.222	1.296	2.552	0.908
Index ΔSUV_max_	0.7	18.3	0.971	1.196	4.299	0.964
Index SUV_peak_1	21.5	15.4	0.163	1.443	3.250	0.892
Index ΔSUV_peak_	−2.4	17.4	0.888	0.874	0.874	0.885
Patient-Level						
qSUV_max_1	17.6	19.2	0.359	1.321	2.759	0.908
ΔqSUV_max_	0.9	27.2	0.972	1.341	4.186	0.935
qSUV_peak_1	36.9	18.3	**0.044**	1.646	3.191	0.840
ΔqSUV_peak_	−15.4	18.8	0.413	1.003	1.609	0.999
qSUV_total_1	0.7	20.9	0.972	2.911	6.061	0.753
ΔqSUV_total_	14.1	25.4	0.580	0.635	1.396	0.794
qVF1	−11.2	21.6	0.606	1.977	4.017	0.808
ΔqVF	15.7	21.2	0.458	0.708	1.063	0.783

^†^ The multivariate model used age, ln(BAP) and the PET parameter. For association with PFS multiple linear regression was used as the data were not censored. PFS days are the number of days corresponding to a 1-SD increase in the PET parameter, and SE is the standard error of Days. Cox proportional hazard modeling was used to determine association of the multivariate model to OS, where 4 patients were censored. The hazard ratio (HR) is the associated hazard ratio corresponding to a 1-SD increase in the PET parameter. The lesion-level analyses were performed on 17 patients at baseline (indicated by 1 after the parameter), while change on-dasatinib was determined on 14 of the 17 patients. The patient-level whole-body QTBI analyses were performed on 16 patients at baseline, while change was determined on 12 of the 16 patients. Boldface type indicates a significant (*p* ≤ 0.05) association with outcome.

**Table 5:** Correlations between change of NaF PET parameters and change in biomarkers.

^†^ PET Parameter	ΔuNTX	ΔBAP	ΔPSA
ΔSUV_maxavg_	0.31	**−0.41**	0.08
	(0.142)	**(0.047)**	(0.747)
ΔSUV_peakavg_	0.26	**−0.45**	0.12
	(0.221)	**(0.026)**	(0.591)
ΔIndex SUV_max_	0.44	−0.21	0.14
	**(0.037)**	(0.331)	(0.518
ΔIndexSUV_peak_	0.23	−0.36	−0.01
	(0.270)	(0.080)	(1.00)
ΔqSUV_max_	0.11	0.00	0.15
	(0.630)	(1.000)	(0.545)
ΔqSUV_peak_	0.02	−0.27	0.12
	(0.945)	(0.250)	(0.638)
ΔqSUV_total_	−0.17	0.03	0.42
	(0.450)	(0.947)	(0.063)
ΔqVF	0.17	−0.06	0.39
	(0.450)	(0.841)	(0.086)

^†^ Kendall tau β rank correlation values (and *p*-values) between the change of NaF PET parameters and the change in PSA and bone biomarkers. Significant correlation of *p*-values (*p* ≤ 0.05) appear in boldface type.

## Data Availability

PET image extracted data for individual patients appears in supplementary martials located on the MDPI website.

## Supplementary Materials

The following are available online at https://www.mdpi.com/article/10.3390/tomography7020013/s1, Figure S1: Study design for ACRIN 6687. ^18^F-fluoride PET was obtained at baseline before therapeutic introduction of dasatinib and at 12 ± 4 weeks into therapy. ^†^ Nilutamide-only patients are not eligible. Patients must be receiving dasatinib to be eligible. However, if a nilutamide patient crosses over at progression to add dasatinib, he may be eligible, Figure S2: The acquisition times for whole-body scans for each patient are represented by lines to indicate the variation in start time and duration of imaging of each scan for the ACRIN 6687 multi-center clinical trial. Paired lines of the same color represent the baseline scan (left) and the on-dasatinib scan (right) imaged 12 weeks later. Some patients had a different number of FOV between their two scans, Figure S3: 74-year-old patient with heterogeneous bone lesions imaged before and after dasatinib. Red box indicates the single dynamic FOV from prior report. Baseline PSA was relatively stable (pre-157, post 185) following 6 cy dasatinib (PFS was 3.0 mos). Arrows indicate the 5 hottest lesions in units of SUV_max_. None of the 5 hottest lesions were assessed in the initial dynamic single FOV imaging study, Figure S4: The same patient described in Figure 1 using the Quantitative Total Bone Imaging (QTBI) analysis software with tumor regions outlined in red. Briefly, CT images were segmented into skeletal regions using an atlas-based approach, then region-specific optimized thresholds were used to detect lesions on the PET image segmentation A random forest model and manual review were applied to exclude lesions that were likely to be benign. The response assessment following dasatinib stratified changes in tumor uptake based off of repeatability measures (Lin 2016), Figure S5: Actual versus predicted time to progression based on multivariate regression analysis. The predicted progression model has age and baseline ln(BAP) and adds in PET SUV_peak_ as covariates. The line shows the standard deviation used for assessment of the correlation (ρ = 0.83) between true and predicted progression and determining hazard ratios. The correlation is highly significant (*p*-value = 0.001), Table S1: The individual PET scanning characteristics are listed for all 18 enrolled patients in the study. Case 5 chose to withdraw from the study after the first scan. Three cases (3, 12 and 16) that progressed early, did not receive the second scan. Uptake time (UptakeT), the time between dose injection and WB scanning, is a sensitive parameter in the assessment of SUV from PET scans. The difference between the uptake times (ΔUT) shows the consistency in the protocol for the scanning institution. The iterative reconstructed image resolution for in-plane X/Y pixel size and slice thickness appears in the last two columns The BLUE highlighted cases are those reported in the original publication using the dynamic PET data. SD is the standard deviation of the group, Table S2: SUV_peakavg_ and SUV_maxavg_ value is the average of up to the 5 hottest tumors that were above the threshold of 15 g/mL in the first scan. Only two patients had less than 5 tumors that met threshold criteria; Case 9 had one tumor and Case 13 had 2 tumors above the threshold. The index SUV is the hottest lesion for the patient. SD is the standard deviation of the group. Case 5 withdrew from the study, so was lost to follow-up for PFS and OS assessment, Table S3: Patient-level analysis results using QTBI software (AIQ Solutions, Inc.) for (A) scan 1 and (B) scan 2. SD is the standard deviation of the group. Case 15 had image quality and scaling issues that prevented analysis. Case 5 withdrew from the study, so was lost to follow-up for PFS and OS assessment, Table S4: Gaussian approximations for inferences using a Bootstrap approach with 500 replicates was used in univariate outcome analysis The change (Δ) while on-dastinib was determined on 14 of the 17 patients. The PFS section has correlation (tau), standard error (SE), and the *p*-value for each PET parameter. The OS section has the hazard ratio (HR), standard error (SE) of HR and the *p*-value for each PET parameter. In the analysis of overall survival, HR has the associated hazard ratio corresponding to a 1-SD increase in the PET parameter. The lesion-level analyses were performed on the average of up to 5 tumors per patient selected by uptake intensity for 17 patients at baseline. The patient-level whole-body QTBI analyses were performed on 16 patients at baseline, while change was determined on 12 of the 16 patients. Boldface type indicates a significant (*p* ≤ 0.05) association with outcome.
