# Supplementary material for: Whole-Body [18F]-Fluoride PET SUV Imaging to Monitor Response to Dasatinib Therapy in Castration-Resistant Prostate Cancer Bone Metastases: Secondary Results from ACRIN 6687
Source: Tomography. 2021 Apr 25;7(2):139–53. doi: 10.3390/tomography7020013 (PMC8167705; doi:10.3390/tomography7020013)
Supplement: Supplementary file 1 [file tomography-07-00013-s001.zip › tomography-1169513 - supplementary - final.pdf]

Whole-body [18F]-fluoride PET SUV imaging to monitor response to dasatinib therapy in castration-resistant prostate cancer bone metastases: secondary results from ACRIN 6687

Mark Muzi[1], Finbarr O'Sullivan[2], Timothy Perk[3], John P. Muzi[1], David A. Mankoff[4], Robert Jeraj[5], Fenghai Duan[6], Evan Y. Yu[1].

Supplemental Materials: Figures and Tables for ACRIN 6687 Secondary Analysis

Additional information is supplied on the study design (Figure S1), a plot of the WB scan duration (Figure S2), an additional example patient that had no tumors overlapping between the dynamic scan and the WB scan based on the largest SUV_{max} (Figure S3), a patient example QTBI patient-level analysis (Figure S4) and a plot of actual PFS vs multivariate model predicted PFS (Figure S5). Tables include a table of individual patient NaF PET scanning parameters (Table S1), a table of individual patient PET SUV metrics from VOI analysis (Table S2), individual patient level measurement from QTBI analysis software (AIQ Solutions, Inc) in Table S3, and univariate statistical analysis results using a bootstrapping approach (Table S4).

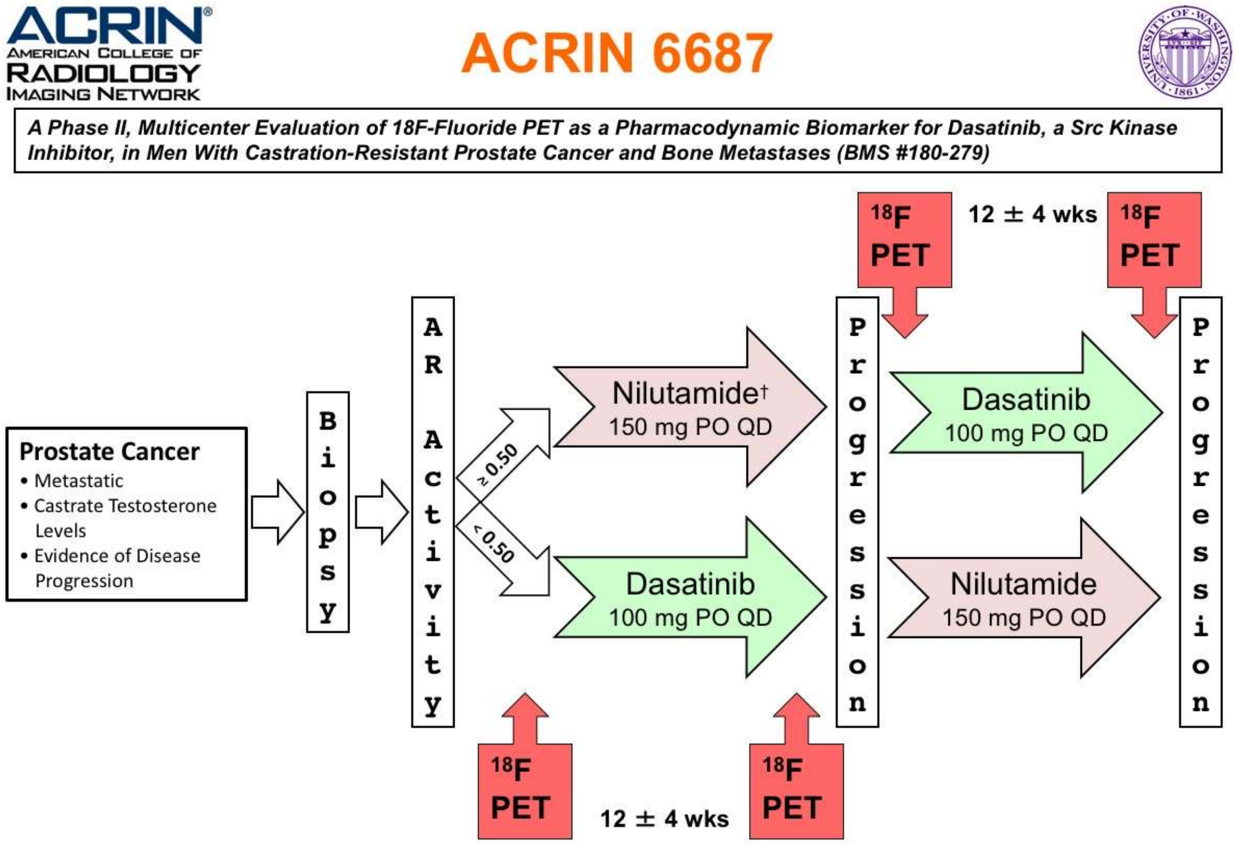

Figure S1. Study design for ACRIN 6687. ^{18}F-fluoride PET was obtained at baseline before therapeutic introduction of dasatinib and at 12 ± 4 weeks into therapy. † Nilutamide-only patients are not eligible. Patients must be receiving dasatinib to be eligible. However, if a nilutamide patient crosses-over at progression to add dasatinib, he may be eligible.

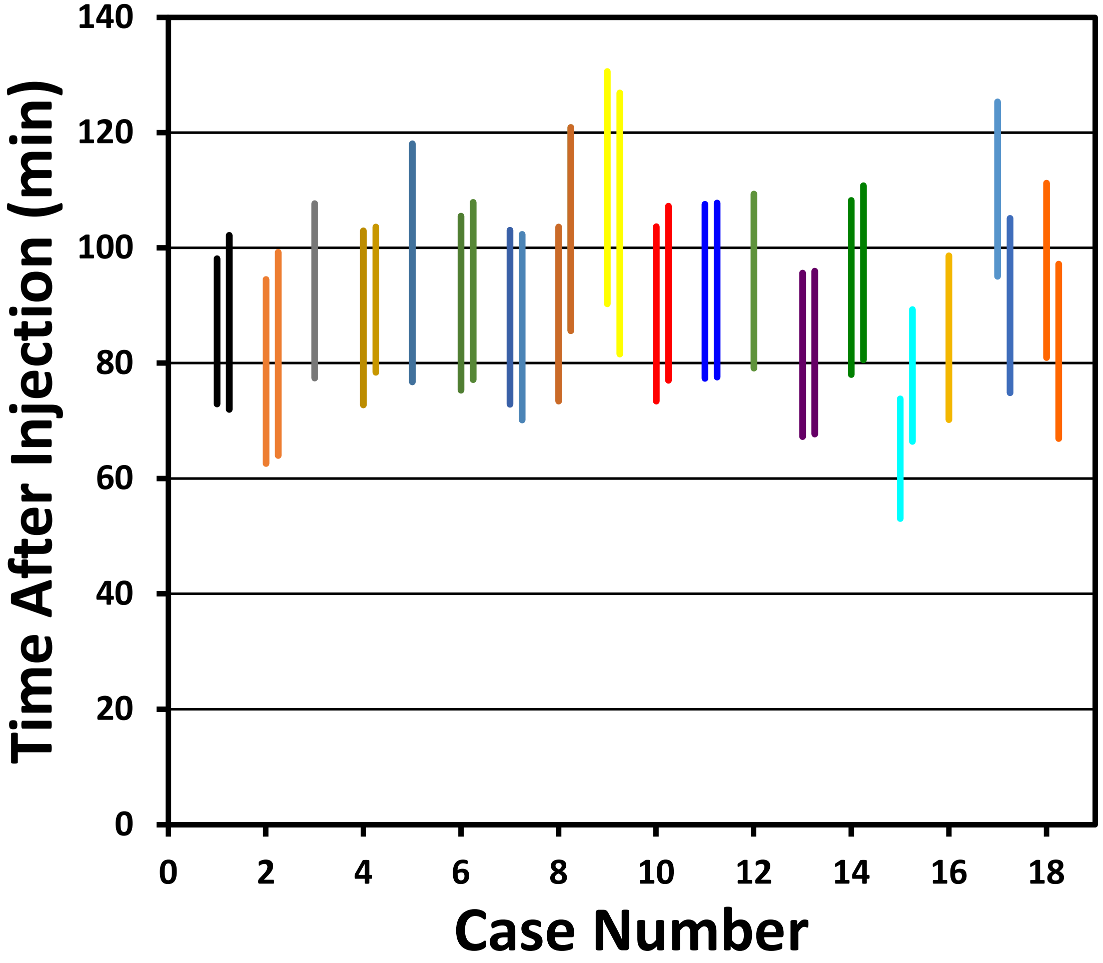

Figure S2. The acquisition times for whole-body scans for each patient are represented by lines to indicate the variation in start time and duration of imaging of each scan for the ACRIN 6687 multi-center clinical trial. Paired lines of the same color represent the baseline scan (left) and the on-dasatinib scan (right) imaged 12 weeks later. Some patients had a different number of FOV between their two scans.

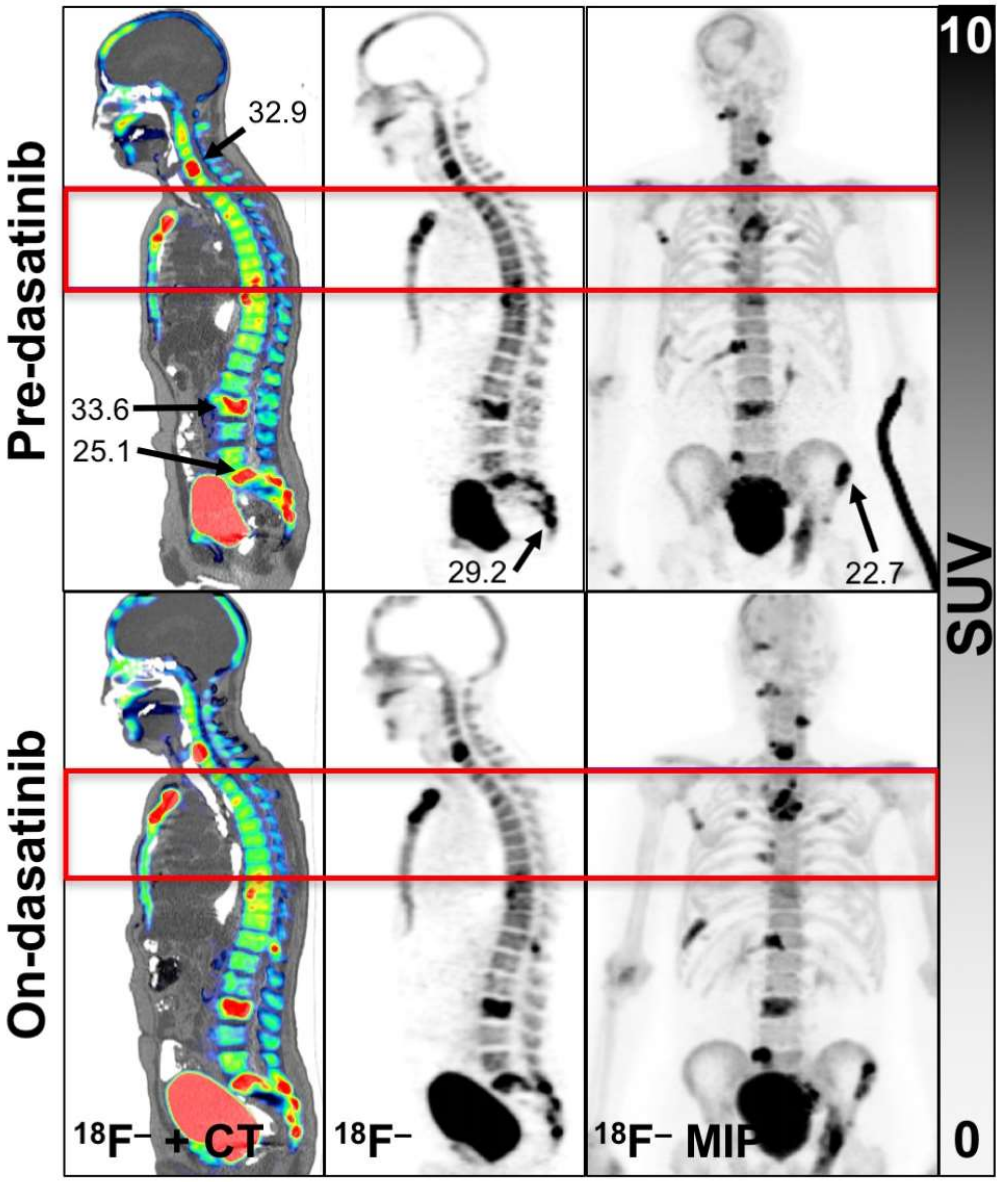

Figure S3. 74-year-old patient with heterogeneous bone lesions imaged before and after dasatinib. Red box indicates the single dynamic FOV from prior report. Baseline PSA was relatively stable (pre-157, post 185) following 6 cy dasatinib (PFS was 3.0 mos). Arrows indicate the 5 hottest lesions in units of SUVmax. None of the 5 hottest lesions were assessed in the initial dynamic single FOV imaging study.

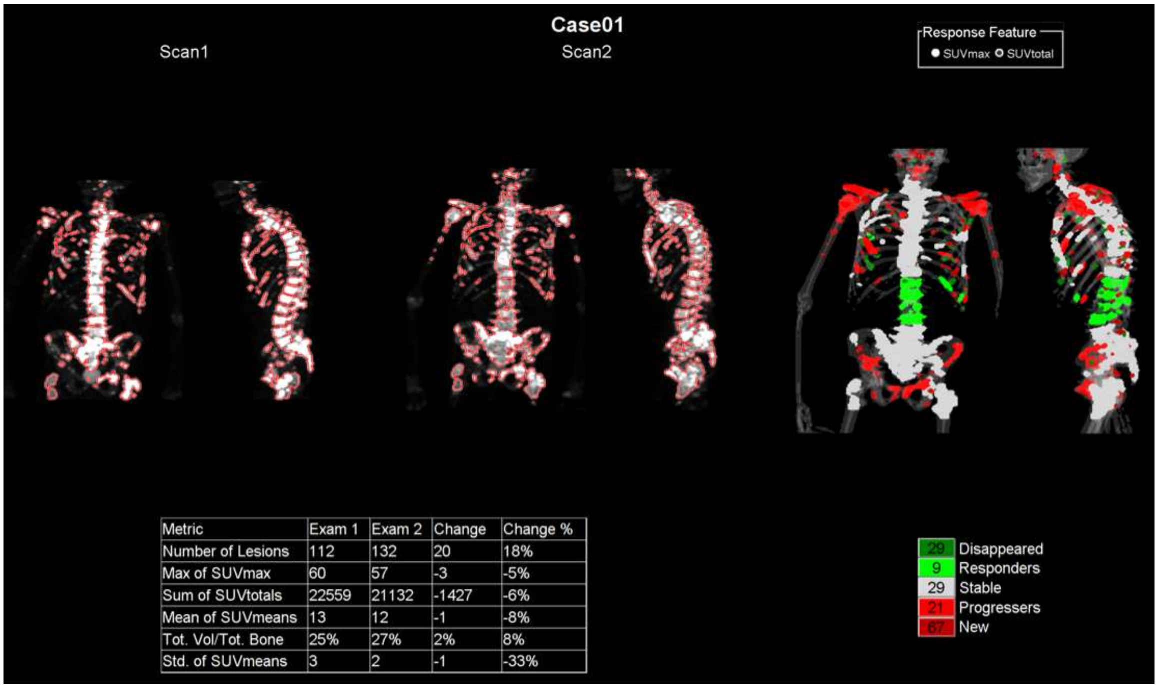

Figure S4. The same patient described in Figure 1 using the Quantitative Total Bone Imaging (QTBI) analysis software with tumor regions outlined in red. Briefly, CT images were segmented into skeletal regions using an atlas-based approach, then region-specific optimized thresholds were used to detect lesions on the PET image segmentation A random forest model and manual review were applied to exclude lesions that were likely to be benign. The response assessment following dasatinib stratified changes in tumor uptake based off of repeatability measures (Lin 2016).

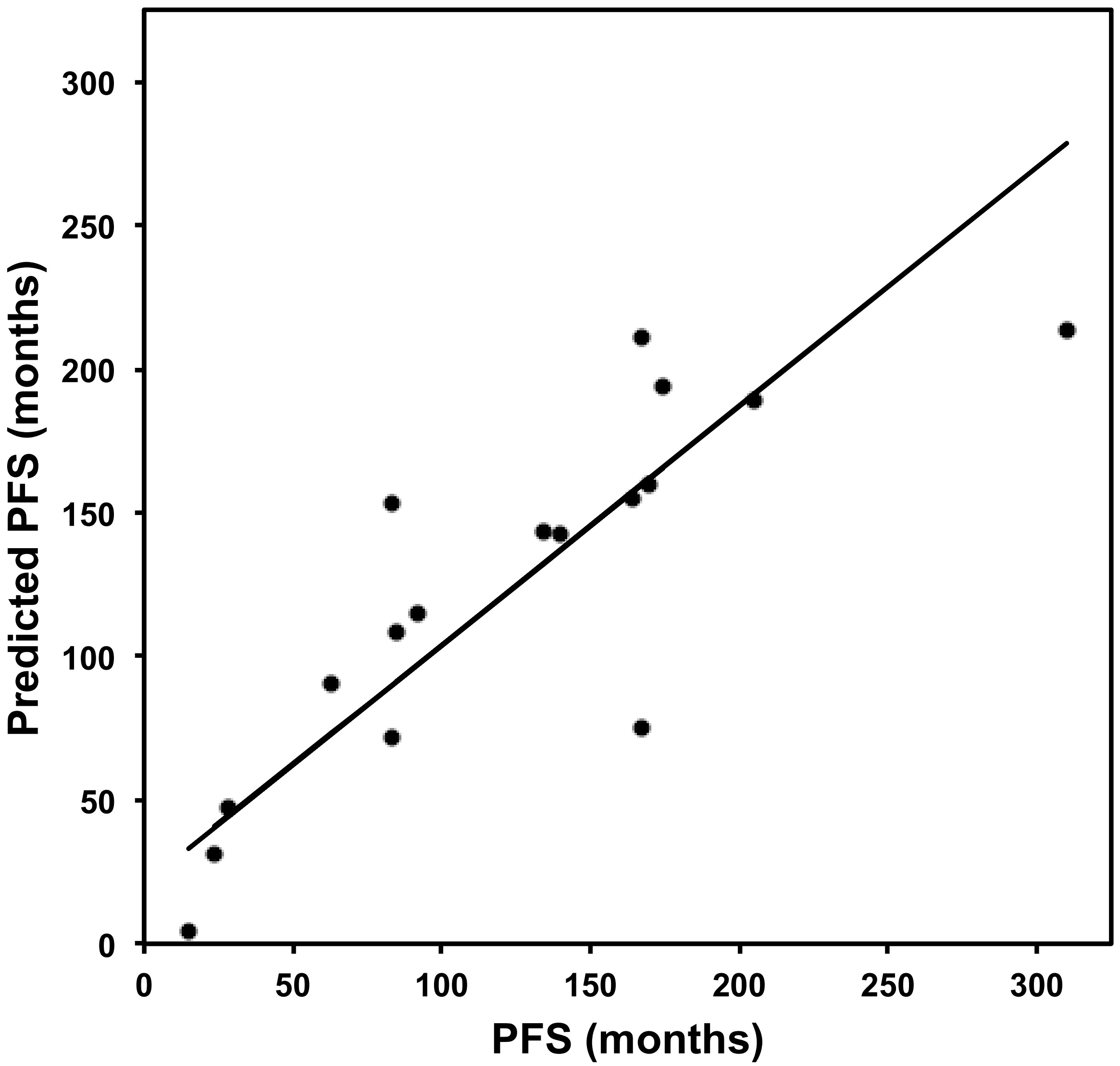

Figure S5. Actual versus predicted time to progression based on multivariate regression analysis. The predicted progression model has age and baseline ln(BAP) and adds in PET SUVpeak as covariates. The line shows the standard deviation used for assessment of the correlation (ϱ = 0.83) between true and predicted progression and determining hazard ratios. The correlation is highly significant (*p*-value = 0.001).

Table S1. The individual PET scanning characteristics are listed for all 18 enrolled patients in the study. Case 5 chose to withdraw from the study after the first scan. Three cases (3, 12 and 16) that progressed early, did not receive the second scan. Uptake time (UptakeT), the time between dose injection and WB scanning, is a sensitive parameter in the assessment of SUV from PET scans. The difference between the uptake times (ΔUT) shows the consistency in the protocol for the scanning institution. The iterative reconstructed image resolution for in-plane X/Y pixel size and slice thickness appears in the last two columns The BLUE highlighted cases are those reported in the original publication using the dynamic PET data. SD is the standard deviation of the group.

Individual Patient PET scan information								
		[18F] Imaging Parameters						
**Case**	**Age**	**Dose1**	**Dose2**	**UptakeT1**	**UptakeT2**	**ΔTime**	**Pixel X/Y**	**Slice**
	**(years)**	**MBq**	**MBq**	**(min)**	**(min)**	**(min)**	**(mm)**	**(mm)**
**1**	83	334	365	73	72	1	4.3	4.3
**2**	74	346	318	63	64	1	4.3	4.3
**3**	79	326		77			4.3	4.3
**4**	79	307	335	73	78	6	4.3	4.3
**5**	48	382		77			3.9	3.3
**6**	66	308	329	75	77	2	4.3	4.3
**7**	86	368	325	73	70	3	4.3	4.3
**8**	75	304	282	73	86	12	4.3	4.3
**9**	85	345	369	90	82	9	3.9	3.3
**10**	67	293	351	73	77	4	4.3	4.3
**11**	66	333	338	77	78	1	4.3	4.3
**12**	57	330		79			4.3	4.3
**13**	70	333	347	67	68	1	4.0	5.0
**14**	79	291	343	78	81	3	4.3	4.3
**15**	62	326	343	53	66	13	4.0	4.0
**16**	51	345		70			4.0	4.0
**17**	64	292	328	95	75	20	4.3	4.3
**18**	60	324	286	81	67	14	4.3	4.3
**Average**	**71**	**324**	**333**	**75**	**74**	**6.4**	**4.2**	**4.2**
**SD**	**10**	**22**	**25**	**9**	**7**	**6.1**	**0.2**	**0.4**

Table S2. $SUV_{peakavg}$ and SUV_{maxavg} value is the average of up to the 5 hottest tumors that were above the threshold of 15 g/mL in the first scan. Only two patients had less than 5 tumors that met threshold criteria; Case 9 had one tumor and Case 13 had 2 tumors above the threshold. The index SUV is the hottest lesion for the patient. SD is the standard deviation of the group. Case 5 withdrew from the study, so was lost to follow-up for PFS and OS assessment.

**Case**	**Lesions**	**WB PET SUV Measurements**					
		**$SUV_{peakavg1}$**	**$SUV_{peakavg2}$**	**$SUV_{maxavg1}$**	**$SUV_{maxavg2}$**	**$SUV_{maxIndex1}$**	**$SUV_{maxIndex2}$**
	**(n)**	**(g/mL)**	**(g/mL)**	**(g/mL)**	**(g/mL)**	**(g/mL)**	**(g/mL)**
**1**	5	45.8	25.5	57.6	34.1	60.2	54.6
**2**	5	20.1	19.6	28.7	28.2	33.6	41.2
**3**	5	25.3		35.9		41.2	
**4**	5	24.0	21.4	32.0	26.5	35.0	33.8
**5**	5	65.9		87.0		97.4	
**6**	5	31.8	25.7	47.2	37.1	96.1	68.2
**7**	5	25.6	20.4	34.5	25.8	40.9	31.6
**8**	5	33.4	45.3	59.3	64.7	78.7	97.4
**9**	1	17.8	3.2	29.7	5.4	29.7	5.4
**10**	5	51.9	34.4	74.0	47.0	111.2	78.7
**11**	5	47.3	42.9	67.1	57.5	94.7	70.6
**12**	5	29.8		38.0		42.1	
**13**	2	51.5	47.1	58.5	52.7	72.7	62.2
**14**	5	44.9	37.3	58.5	50.5	78.7	69.4
**15**	5	17.2	18.7	25.3	23.7	27.5	27.5
**16**	5	45.9		58.4		60.1	
**17**	5	54.3	43.1	70.2	57.6	86.9	66.6
**18**	5	19.7	19.2	26.6	25.5	30.3	31.2
**Average**	**4.6**	**34.5**	**28.8**	**47.1**	**38.3**	**60.0**	**52.8**
**SD**	**1.2**	**13.3**	**13.0**	**16.7**	**17.0**	**27.3**	**24.9**

Table S3. Patient-level analysis results using QTBI software (AIQ Solutions, Inc) for (A) scan 1 and (B) scan 2. SD is the standard deviation of the group. Case 15 had image quality and scaling issues that prevented analysis. Case 5 withdrew from the study, so was lost to follow-up for PFS and OS assessment.

(A)

Case	Lesions1	QTBI WB PET SUV Measurements: Scan 1				
		qSUVpeak1	qSUVmax1	qSUVmean1	qSUVtotal1	qVF1
#	(n)	(g/mL)	(g/mL)	(g/mL)	(g/mL)	(%)
1	112	45	60.2	12.7	22559	25%
2	14	20	32.1	13.5	1222	2%
3	114	25	41.2	11.9	5815	9%
4	74	24	35.0	10.9	16653	27%
5	70	66	96.6	17.0	9936	7%
6	26	33	96.1	13.6	1686	3%
7	110	20	31.6	11.4	2923	5%
8	78	35	78.7	13.7	3115	4%
9	11	12	29.7	11.7	99	0%
10	31	51	111.2	16.3	18007	16%
11	26	48	94.7	16.0	2281	3%
12	74	29	42.1	12.5	17663	21%
13	1	65	72.7	25.5	4807	4%
14	31	46	78.7	15.9	2648	2%
15						
16	89	48	60.1	13.5	27397	29%
17	20	54	85.0	21.4	2120	2%
18	79	20	30.3	11.1	2758	5%
**Average**	56	38	63	15	8335	10%
**SD**	38	16	28	4	8641	10%

(B)

Case	Lesions2	QTBI WB PET SUV Measurements: Scan 2				
		qSUVpeak2	qSUVmax2	qSUVmean2	qSUVtotal2	qVF2
#	(n)	(g/mL)	(g/mL)	(g/mL)	(g/mL)	(%)
1	132	37	57.3	12.3	21132	27%
2	23	22	41.2	13.2	1591	2%
3						
4	84	23	35.9	10.5	14162	20%
5						
6	22	29	69.0	13.4	1621	2%
7	130	26	40.9	12.4	4241	7%
8	91	51	97.4	14.8	5575	7%
9						
10	46	50	120.5	13.8	19741	19%
11	29	47	70.6	16.1	2667	3%
12						
13	1	57	62.2	23.7	4490	4%
14	31	43	73.7	15.4	3073	3%
15						
16						
17	33	46	72.9	17.6	4967	4%
18	75	22	31.8	10.7	4425	8%
**Average**	58	38	64	14	7307	9%
**SD**	44	13	26	4	6950	8%

Table S4. Gaussian approximations for inferences using a Bootstrap approach with 500 replicates was used in univariate outcome analysis The change (Δ) while on-dastinib was determined on 14 of the 17 patients. The PFS section has correlation (tau), standard error (SE), and the p-value for each PET parameter. The OS section has the hazard ratio (HR), standard error (SE) of HR and the p-value for each PET parameter. In the analysis of overall survival, HR has the associated hazard ratio corresponding to a 1-SD increase in the PET parameter. The lesion-level analyses were performed on the average of up to 5 tumors per patient selected by uptake intensity for 17 patients at baseline. The patient-level whole-body QTBI analyses were performed on 16 patients at baseline, while change was determined on 12 of the 16 patients. Boldface type indicates a significant ($p \leq 0.05$) association with outcome.

Univariate analysis of PET variables to Outcomes using Bootstrap Analysis						
	**PFS (tau)**	**SE**	**P-value**	**OS (HR)**	**SE**	***p*-value**
Lesion-Level						
SUVmaxavg1	0.061	0.150	0.683	1.292	0.468	0.533
ΔSUVmaxavg	−0.046	0.174	0.793	0.808	2.426	0.937
SUVpeakavg1	0.062	0.170	0.716	1.388	0.547	0.478
ΔSUVpeak	−0.173	0.172	0.314	0.790	0.260	0.421
Index SUVmax1	0.005	0.165	0.977	1.196	0.502	0.697
Index ΔSUVmax	−0.064	0.202	0.751	0.896	2.431	0.966
IndexSUVpeak1	−0.010	0.166	0.950	1.224	0.430	0.602
Index ΔSUVpeak	0.046	0.196	0.816	0.761	0.457	0.601
Patient-Level						
qSUVmax1	−0.091	0.186	0.624	1.187	0.496	0.706
ΔqSUVmax	−0.028	0.249	0.909	0.884	0.381	0.761
qSUVpeak1	0.026	0.210	0.901	1.510	0.743	0.492
ΔqSUVpeak	0.004	0.212	0.984	0.826	0.895	0.846
qSUVtotal1	−0.279	0.188	0.137	2.320	2.723	0.628
ΔqSUVtotal	0.076	0.260	0.772	0.770	2.420	0.924
qVF1	−0.411	0.161	**0.011**	1.908	1.031	0.378
ΔqVF	0.156	0.258	0.545	0.831	1.006	0.867
